# Identification of a laccase Glac15 from *Ganoderma lucidum* 77002 and its application in bioethanol production

**DOI:** 10.1186/s13068-015-0235-x

**Published:** 2015-03-31

**Authors:** Zemin Fang, Xiaoman Liu, Liyuan Chen, Yu Shen, Xuecheng Zhang, Wei Fang, Xiaotang Wang, Xiaoming Bao, Yazhong Xiao

**Affiliations:** School of Life Sciences, Anhui University, 111 Jiulong Road, Hefei, Anhui 230601 China; Anhui Provincial Engineering Technology Research Center of Microorganisms and Biocatalysis, 111 Jiulong Road, Hefei, Anhui 230601 China; The State Key Laboratory of Microbial Technology, School of Life Sciences, Shandong University, 27 Shanda Nanlu, Jinan, Shandong 250100 China; Department of Chemistry & Biochemistry, Florida International University, Miami, FL 33199 USA

**Keywords:** Laccase, *Ganoderma lucidum*, Identification, Characterization, Bioethanol, Detoxification

## Abstract

**Background:**

Laccases have potential applications in detoxification of lignocellulosic biomass after thermochemical pretreatment and production of value-added products or biofuels from renewable biomass. However, their application in large-scale industrial and environmental processes has been severely thwarted by the high cost of commercial laccases. Therefore, it is necessary to identify new laccases with lower cost but higher activity to detoxify lignocellulosic hydrolysates and better efficiency to produce biofuels such as bioethanol. Laccases from *Ganoderma lucidum* represent proper candidates in processing of lignocellulosic biomass.

**Results:**

*G. lucidum* 77002 produces three laccase isoenzymes with a total laccase activity of 141.1 U/mL within 6 days when using wheat bran and peanut powder as energy sources in liquid culture medium. A new isoenzyme named Glac15 was identified, purified, and characterized. Glac15 possesses an optimum pH of 4.5 to 5.0 and a temperature range of 45°C to 55°C for the substrates tested. It was stable at pH values ranging from 5.0 to 7.0 and temperatures lower than 55°C, with more than 80% activity retained after incubation for 2 h. When used in bioethanol production process, 0.05 U/mL Glac15 removed 84% of the phenolic compounds in prehydrolysate, and the yeast biomass reached 11.81 (optimal density at 600 nm (OD_600_)), compared to no growth in the untreated one. Addition of Glac15 before cellulase hydrolysis had no significant effect on glucose recovery. However, ethanol yield were improved in samples treated with laccases compared to that in control samples. The final ethanol concentration of 9.74, 10.05, 10.11, and 10.81 g/L were obtained from samples containing only solid content, solid content treated with Glac15, solid content containing 50% prehydrolysate, and solid content containing 50% prehydrolysate treated with Glac15, respectively.

**Conclusions:**

The *G. lucidum* laccase Glac15 has potentials in bioethanol production industry.

**Electronic supplementary material:**

The online version of this article (doi:10.1186/s13068-015-0235-x) contains supplementary material, which is available to authorized users.

## Background

Bioethanol production from renewable biomass such as lignocellulosic materials has been the focus of numerous contemporary investigations with the decreasing supply of fossil fuels and the increasing environmental concerns associated with them [1]. Lignocellulosic feed stocks such as sugar cane bagasse and corn stover are cheap, renewable, and abundant in nature. They do not compete with food production and contribute to reduce the use of fossil fuels, thus alleviating carbon dioxide emission and global warming [1]. Lignocellulosic feed stocks mainly consist of two polysaccharides, cellulose and hemicellulose, which can be hydrolyzed to provide monosaccharides used by microbial biocatalysts in fermentation processes [2,3]. Unfortunately, cellulose and hemicellulose are closely linked by lignin, a polymer that acts as cementing agent between cellulose fibers [2]. Lignocellulosic materials are thus recalcitrant to hydrolysis and require pretreatment before being converted to bioethanol [3,4].

The combination of steam explosion and acidic catalyst such as H_2_SO_4_ or SO_2_ is one of the most commonly used methods for lignocellulose pretreatment, since it breaks and/or removes a minor part of lignin, depolymerizes cellulose and hemicellulose, and makes the biomass more accessible to hydrolytic enzymes with relatively lower cost [5]. However, this pretreatment generates some soluble inhibitory compounds from partial degradation of sugars and lignin, which can affect enzymatic hydrolysis as well as fermentation steps, and will reduce the ethanol productivity of the microorganisms and the final ethanol yield [2,3,5].

The inhibitory compounds generated during the pretreatment process include weak acids, furan derivatives, and phenolic and other compounds [2]. Several procedures for the removal of these compounds including biological, physical, and chemical methods have been assayed. Among which, enzymatic treatment using laccases has been suggested as one of the most promising approach in lignocellulosic biomass detoxification [3,5]. Laccases (benzenediol:oxygen oxidoreductases, EC1.10.3.2) are a family of blue multicopper oxidases that are capable of oxidizing a wide range of phenolic and aromatic compounds, with concomitant reduction of molecular oxygen to water [6]. When detoxifying the lignocellulosic hydrolysate, laccase was suggested to be selective and can virtually remove all phenolic monomers. Compared with other approaches, advantages of using laccase are that enzyme preparations have higher catalytic efficiencies, fewer toxic subproducts, shorter treatment time, and lower energy costs [3,5,7]. The disadvantage of using laccase is the high enzyme production costs, as the commercial laccases are still expensive despite strides made to reduce enzyme cost through modern biotechnology, thus restricting the applications of laccases [3]. Screening for laccases with higher lignocellulosic hydrolysate-detoxifying ability may improve the present situation.

Laccases are widespread in white-rot fungi. Among which, *Ganoderma lucidum* is a representative that is able to grow on withered wood and other agro-lignocellulosic biomass materials and can decompose lignin by secreting enzymes such as laccases [8]. For instance, *G. lucidum* is one of the richest set of wood degradation enzymes among all of the sequenced basidiomycetes [9]. Based on this, and the fact that *G. lucidum* can produce high amount of laccase in liquid cultures [10-12], laccases from this fungus may promote degradation/modification of lignin or lignin-derived components [13] and therefore represent proper candidates in processing lignocellulosic biomass. To address this issue, a fungal strain *G. lucidum* 77002 was investigated for laccase production by using agricultural by-products as media, and a laccase identified as Glac15 was purified and characterized for the first time; its application potential in lignocellulosic biomass detoxification after thermochemical pretreatment was also evaluated. Our results indicated that Glac15 is a promising candidate in bioethanol production.

## Results and discussion

### Laccase production in liquid culture

**Figure 1:**
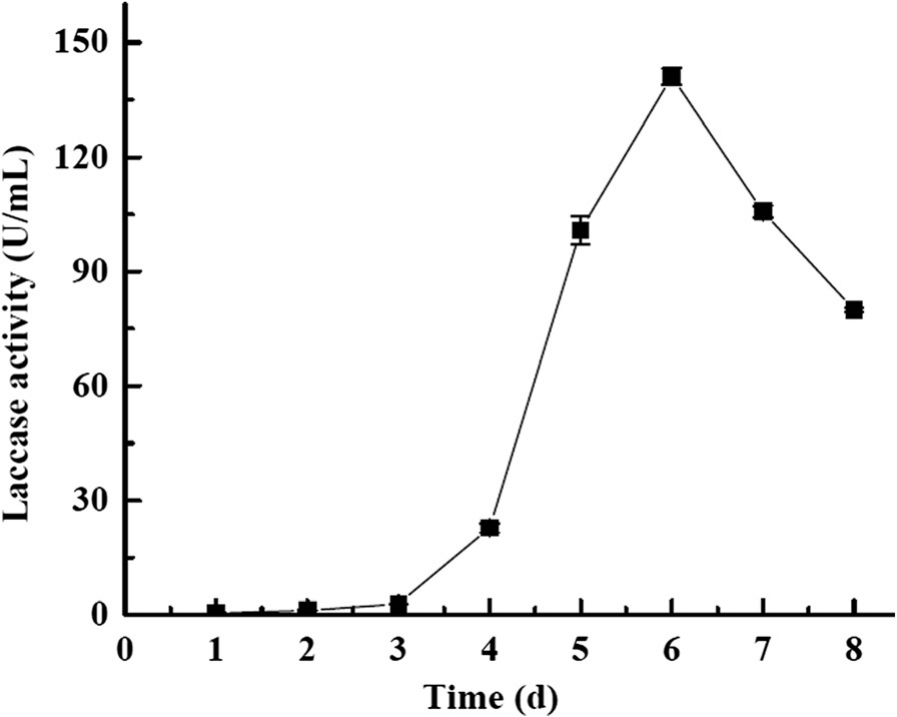
**Laccase activity of the** ***G. lucidum*** **using wheat bran and peanut powder in liquid culture.**

**Figure 2:**
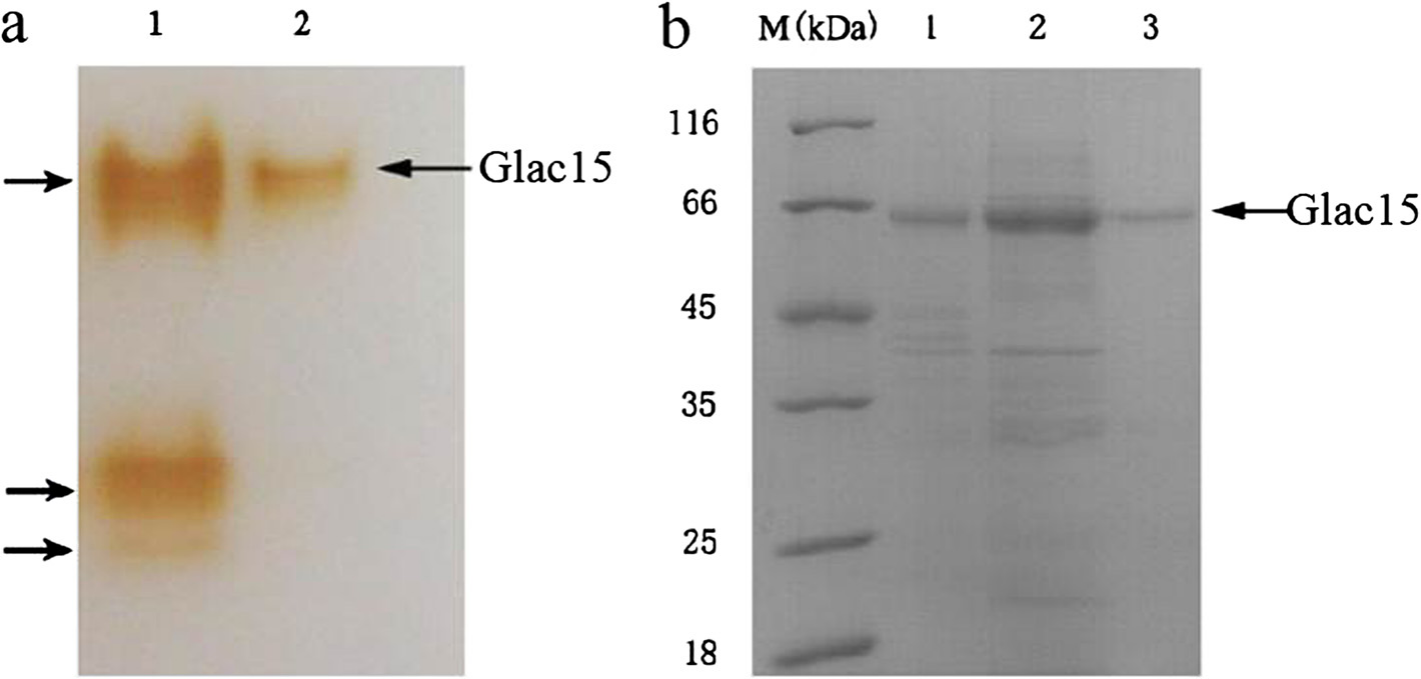
**Native PAGE and SDS-PAGE of laccase purification. (a)** Zymography of crude and purified laccase. Laccase activity was performed using 1 mM guaiacol as substrate. Lanes: 1: crude laccase; 2: purified laccase. **(b)** SDS-PAGE of crude and purified laccase. Lanes: 1: crude protein extract produced by filtering the fermentation supernatant through six layers of sterile gauze; 2: crude protein extract after ultrafiltration; 3: purified Glac15 after DEAE-Sepharose FF chromatography.

Wheat and peanut are two of the most common agricultural crops in China. To obtain laccases with lower cost, along with the fact that wheat bran can induce high amount of lignocellulose-degrading enzymes including laccase in liquid culture [14], wheat bran (3% dry weight, *w*/*v*) and peanut powder (3% dry weight, *w*/*v*) were used as energy sources to induce *G. lucidum* 77002 to produce laccase. Laccase activity reached 141.1 ± 0.2 U/mL within 6 days when assayed using 2,2′-azino-bis-(3-ethylbenzothiazoline-6-sulfonic acid) (ABTS) as a substrate (Figure 1), indicating that these agriculture by-products could effectively induce laccase production. This is consistent with previous reports that other agriculture and forest by-products such as pine and poplar ground woods [10] and wheat bran [14] can induce laccase production in *G. lucidum*, which were 0.93 and 97.34 U/mL, respectively. Besides, tamarind shell with ethanol (3%, *v*/*v*), CuSO_4_ (0.4 mM), and gallic acid (1 mM)-containing medium can also induce the *G. lucidum* laccase production, yielding about 74.84 U/mL laccase activity after 15 days, which was 416 times higher than the control [15]. However, a different laccase zymography was shown according to culture conditions. As indicated by native polyacrylamide gel electrophoresis (PAGE), *G. lucidum* 77002 produced three laccase isoforms in this study (Figure 2a), whereas a *G. lucidum* strain produced only two laccase isoenzymes in high-nitrogen culture and in cultures containing both poplar and pine [10]. In contrast to these results, when induced by the mixture of tamarind shell, ethanol, CuSO_4_, and gallic acid, another *G. lucidum* strain produced four laccase isoenzymes [15]. Responsive elements that are found in various locations along the laccase promoter sequences may contribute to the variation in the laccase isoforms produced among *G. lucidum* strains. Several reports have highlighted their presence and postulated that these responsive elements, including metal-responsive element, xenobiotic response element, heat shock-responsive element, and antioxidant response element, may regulate laccase gene transcription, and their locations and orientations suggest a complex picture of laccase expression regulation [16]. On the other hand, the differential expression of laccase isoenzyme genes might also arise from different ecological origins of these mushrooms [6,8].

### Laccase purification and characterization

Laccase was purified from crude culture filtrate according to the methods described in the ‘Methods’ section. The summary of purification steps is presented in Table 1. One laccase was 14.19-fold purified from its initial culture broth with a final yield of 5.5%. The purified laccase exhibited a single band both on native PAGE and sodium dodecyl sulfate (SDS)-PAGE (Figure 2), suggesting a homogenous form. The specific activity of the laccase was 186 U/mg when estimated with ABTS as the substrate.

**Table 1:** **Summary of the purification steps of extracellular laccase Glac15 from** ***G. lucidum*** **77002**

**Purification step**	**Total volume (ml)**	**Total activity (U)**	**Total Protein (mg)**	**Specific activity (U/mg)**	**Percent recovery (%)**	**Purification fold**
Crude culture filtrate	1,250	47,600	3,630	13.1	100.00	1.00
Ultrafiltration	100	28,000	1,100	25.5	58.8	1.94
DEAE-Sepharose FF	30	2,600	14	186	5.5	14.19

*G. lucidum* contains 16 laccase genes in its genome [17]. Based on data-dependent liquid chromatography*-*electrospray ionization*-*tandem mass spectrometry (LC-ESI-MS/MS), the purified laccase matched the laccases Glac6 and Glac15 (Additional file 1: Table S1 and Table S2) in the genome of *G. lucidum* Xiangnong No.1. Furthermore, its N-terminal sequence was GIGPTTDLTISNADI, completely matching the amino acidic sequence starting at position 22 of Glac15. However, *G. lucidum* 77002 is not the same strain compared to *G. lucidum* Xiangnong No.1, indicating that the purified protein may be Glac15, and suggested that a 21-amino acid signal peptide exists in the zymogen. As the protein obtained in this study showed the highest sequence identity with Glac15 from *G. lucidum* Xiangnong No.1, it was also designated as Glac15. To the best of our knowledge, this is the first report of Glac15 from *G. lucidum* [10-15,17-22]. Data bank homology searches (NCBI) further confirms the novelty of the purified Glac15 (Table 2) and returned a laccase from *G. lucidum* (GenBank: AHA83584) as the best hit (93% identity), followed by another two laccases from *G. lucidum* (GenBank: ABK59822 and ABK59823) with 87% identity. These three proteins were identified by whole genome sequencing and have not been characterized. When compared with the characterized laccases from *G. lucidum*, the N-terminal region of Glac15 shared the highest sequence homology of 93% with the laccase of KULac1, KULac3, KULac4, and KULac5 from *Ganoderma* sp. KU-Alk4 [22] (Table 2).

**Table 2:** **Overview of N-terminal sequence of Glac15 to other laccases from** ***G. lucidum***

**Protein**	**Sequence**	**Reference**
Lac15	GIGPTTDLTISNADI	This study
GLac 1, 2, 3	GIGPT	[18]
-	GQNGDAVP	[19]
-	GIGPK	[20]
GLlac1	GIGPK	[21]
KULac 2	GIGPVADLTVRGGDI	[22]
KULac 1; 3; 4; 5	GIGPVTDLTISNADI	[22]
Lac1	GIGPTTDLTISNANI	AHA83584
Lac4	GIGPKTDLTISNADV	ABK59822/ABK59823

### Effect of pH on Glac15 activity

**Figure 3:**
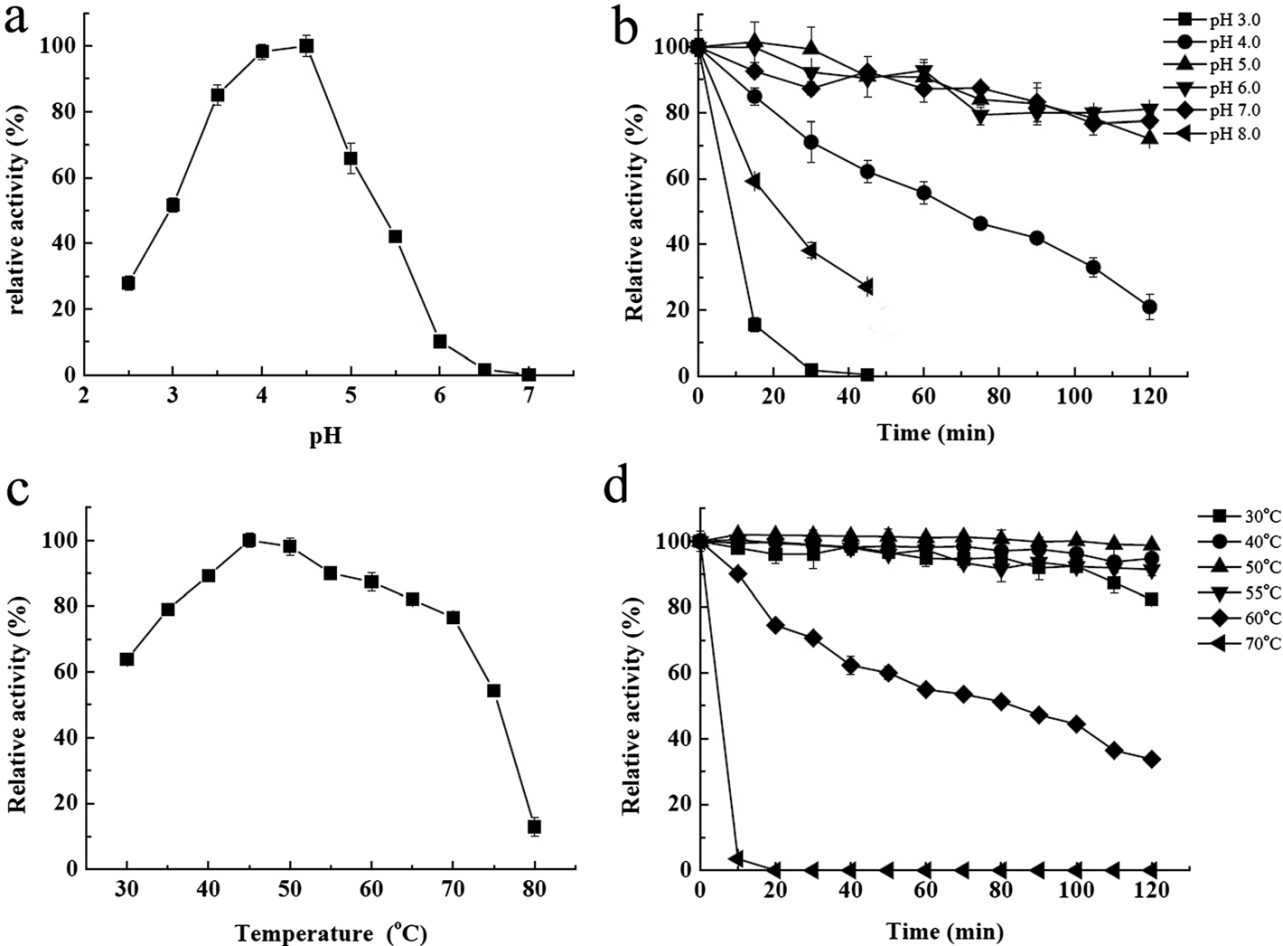
**Effects of pH and temperature on the activity and stability of Glac15. (a)** pH optimum, **(b)** pH stability, **(c)** temperature optimum, and **(d)** thermostability. Laccase activity was determined in 50 mM citrate-Na_2_HPO_4_ buffer.

The pH profiles for fungal laccases toward phenolic substrates are usually narrow bell-shaped, with optimal pH around 5, and little activity can be detected at neutral pH or above [23,24]. Similar to most fungal laccases, Glac15 could oxidize laccase-specific substrate syringaldazine at a pH range of 4.0 to 7.0, with a pH optimum of 4.5 (Figure 3a). The pH optimum for Glac15 on oxidizing other phenol substrates such as 2,6-dimethoxyphenol (2,6-DMP), guaiacol, catechol, and L-dopamine were 4.5, 5.0, 5.0, and 4.5, respectively (Table 3). In contrast, for non-phenolic substrates, ABTS and K_4_Fe(CN)_6_, Glac15 has been observed to present monotonic activity profiles, in which the rate decreased as the pH increased from 2.5 to 7.0, with the optimum pH being 2.5 (Table 3), like most fungal laccases [23]. The monotonic pH-dependent decrease of the activity has been interpreted as the sole effect of hydroxyl ion inhibition [24]. On the other hand, Glac15 was highly stable at pH 5.0 to 7.0. After incubation at 50°C for 120 min, Glac15 retained more than 70% of its original activities. In contrast, the enzyme was unstable at low pH, with approximately 20% activity left after incubation at pH 4.0 (Figure 3b) for 120 min at 50°C. This is similar to most fungal laccases, which are functional at acidic and near neutral pH but lose their activities under alkaline conditions [22,25].

**Table 3:** **Substrate specificity of Glac15**

**Substrate**	**Optimal pH**	**Optimal temp. (°C)**	***K***_**m**_ **(M)**	***k***_**cat**_ **(s** ^**-1**^ **)**	***k***_**cat**_ **/** ***K***_**m**_ **(M** ^**-1**^ **s** ^**-1**^ **)**
Syringaldazine	4.5	45	6.2 × 10^**−**5^	3.87 × 10^3^	6.24 × 10^7^
2,6-DMP	4.5	45	1.2 × 10^**−**4^	7.44 × 10^2^	6.20 × 10^6^
Guaiacol	5.0	55	7.9 × 10^**−**4^	1.70 × 10^3^	2.15 × 10^6^
Catechol	5.0	45	7.8 × 10^**−**4^	5.48 × 10^3^	7.03 × 10^6^
L-dopamine	4.5	50	8.9 × 10^**−**4^	2.95 × 10^3^	3.31 × 10^6^
ABTS	2.5	50	1.9 × 10^**−**5^	2.00 × 10^2^	1.05 × 10^7^
K_4_Fe(CN)_6_	2.5	45	4.6 × 10^**−**3^	2.71 × 10^4^	5.89 × 10^6^

### Effect of temperature on Glac15 activity

Glac15 showed its maximal activity at 45°C toward syringaldazine and displayed more than 80% of the maximal activity at temperatures ranging from 35°C to 65°C for syringaldazine (Figure 3c). However, laccase from *G. lucidum* with the optimal temperature below 35°C has been described, for example, Ko *et al.* [18] reported a *G. lucidum* laccase showed the highest activity at 25°C. The thermostability of fungal laccases varies considerably [23]. In contrast to a *G. lucidum* laccase, which was inactivated at 60°C [18], Glac15 was rather stable at temperatures lower than 55°C, with about 90% activity retained after incubation at pH 5.0 and 55°C for 2 h (Figure 3d). When incubated at 60°C, Glac15 retained 40% activity after 120 min, with a half-life of 82 min (Figure 3d). Compared with other fungal laccases with high thermostability, Glac15 may be more suitable for several specific applications that run in moderate temperature, such as food and drink industries including ethanol production [23,26].

### Substrate specificity

Diverse substrates were oxidized by Glac15, and the kinetic parameters of the enzyme were calculated for each of them. As summarized in Table 3, ABTS and syringaldazine were the most reactive substrates for Glac15 in terms of affinity (*K*_m_) and catalytic efficiency (*k*_cat_/*K*_m_). The *K*_m_ values for ABTS and syringaldazine were in micromolar range. As most fungal laccases, these results confirm the specificity of ABTS and syringaldazine as non-phenolic and phenolic substrates, respectively, in laccase enzyme reaction. Glac15’s substrates range was ranked as follows: ABTS > syringaldazine > 2,6-DMP > catechol > guaiacol > L-dopamine > K_4_Fe(CN)_6_. This is quite different from Tplac from *Trametes pubescens*, which showed a rank of ABTS > 2,6-DMP > L-dopa > guaiacol > syringaldazine > catechol [27]. Meanwhile, the *K*_m_ and *k*_cat_/*K*_m_ values for ABTS were 19 μM and 10.5 M^-**1**^/s^-**1**^, respectively, higher than those of certain fungal laccases [28,29].

### Effects of organic solvents, metal ions, and inhibitors on Glac15 activity

**Figure 4:**
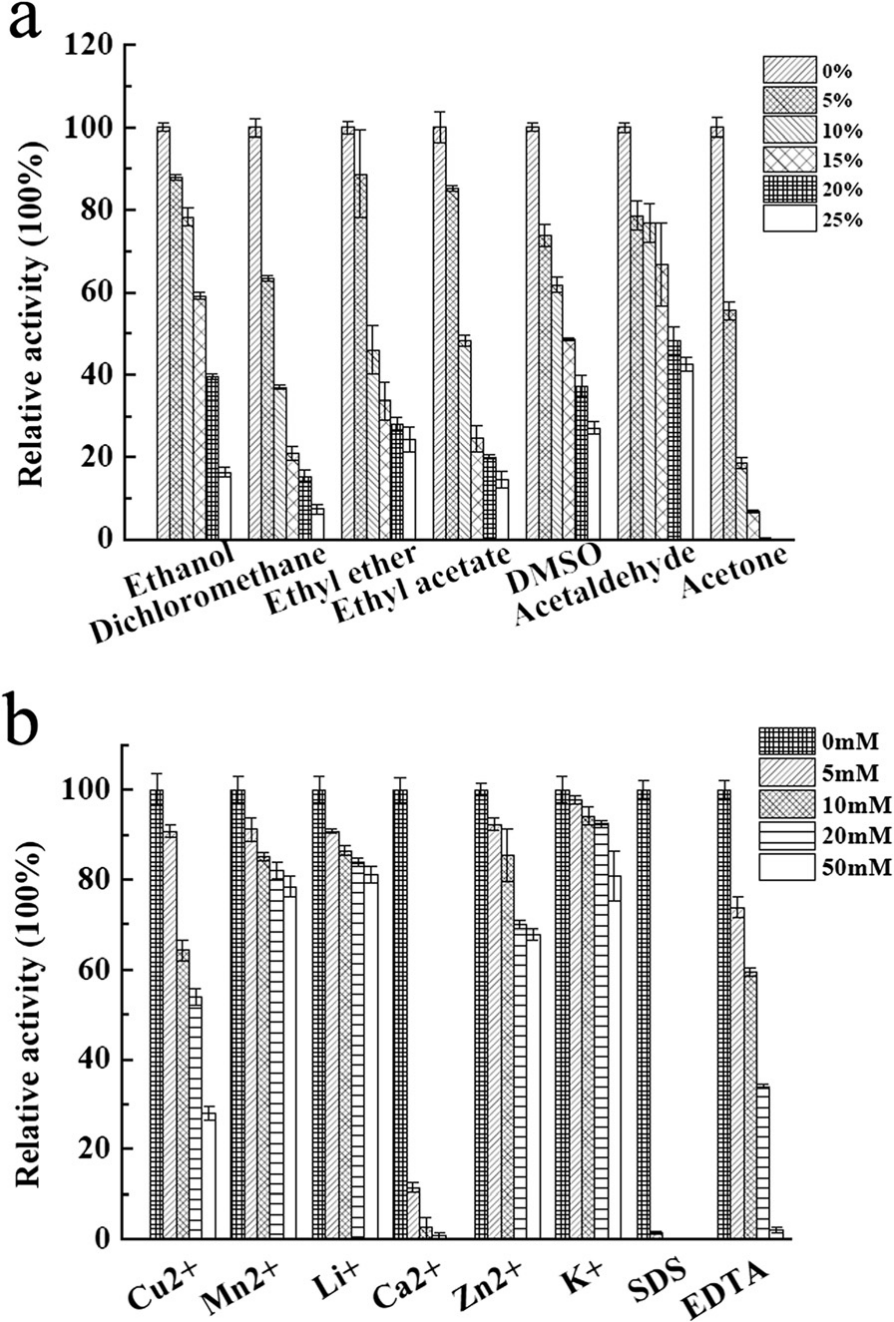
**Effects of organic solvents, metal ions, and inhibitors on Glac15 activity. (a)** Organic solvents and **(b)** metal ions and inhibitors.

Although there are several studies on characterization of laccases from *G. lucidum*, few of them reported the effects of organic solvents on the enzyme activity [10-15,17-22]. In order to obtain more information about the laccase from *G. lucidum*, ethanol, dichloromethane, ethyl ether, ethyl acetate, dimethyl sulfoxide (DMSO), acetaldehyde, and acetone were used to test the effects of organic solvents on Glac15 activity. All of the organic solvents tested inhibited protein activity, with about 80% of the original laccase activity was retained in the presence of 5% organic solvent (*v*/*v*). This inhibitory effect of organic solvents increases as their concentration was increased (Figure 4). This is consistent with previous studies demonstrating that fungal laccases from *Trametes versicolor* [30] and *Pleurotus ostreatus* [31] were inactivated over 10% of the organic solvents. The instability of laccase in organic solvents may be attributed to the denaturation of the enzyme structure caused by interfering hydrophobic effects or the substitution of water molecules by the organic solvents at the active sites [30]. However, Glac15 retained about 60% of its original activity in the presence of 15% ethanol (Figure 4). Enzymatic reactions in compatible organic solvents such as ethanol allow access of laccase to some water-insoluble substrates, which may be helpful in the detoxification of many recalcitrant organic pollutants [32].

Cu^2+^ had negative effect on Glac15 activity (Figure 4). This is apparently different from bacterial laccases, as no activity was detected for the purified bacterial laccase but resumed by addition of Cu^2+^ [33]. Similar to Cu^2+^, Ca^2+^ also inhibited Glac15 activity. Two theories are proposed to explain the metal effects regarding laccase activity. One is that the binding of several metal ions, that is, Cu^2+^ or Ca^2+^, induces conformational modification of the enzyme and stimulates decomposition of the trimer complex containing substrate, enzyme, and metal ion, as evidenced by non-competitive inhibition model. The other is that the metal ion binds near the TI site of laccase and acts as a competitive inhibitor of electron donors by blocking the access of substrates to the TI site or inhibiting the electron transfer at the TI active site, thereby leading to inhibition of laccase activity [27,33]. Other cations such as Mn^2+^, Li^+^, Zn^2+^, and K^+^ at a final concentration of 10 mM did not have noticeable effects on Glac15 activity.

SDS is one of the anionic detergents known for its deleterious effect on the activity of most enzymes. In this study, SDS inhibited the activity, with complete inactivation (about 100%) in 5 mM SDS (Figure 4). According to Moore and Flurkey, the inhibition effect of SDS may be explained by the binding of small amounts of SDS on Glac15 which alters both its enzymatic and physical characteristics [34]. However, only partial inhibition (about 74%) was observed for Glac15 in the presence of 5 mM ethylenediaminetetraacetic acid (EDTA) (Figure 4), compared with complete inactivation observed for laccases from *Thermus thermophilus* [35] and *Aspergillus ochraceus* [36] in the presence of 1 mM EDTA. Cabana *et al.* [37] have reported that the aggregation of laccase as cross-linked enzyme aggregates (CLEAs) improved its stability against EDTA by hindering access of EDTA to the Cu(II) ions of the protein. Thus, the resistance of Glac15 toward EDTA might be ascribed to the low accessibility of EDTA to the structural copper atoms of the protein.

### Laccase treatment of corn stover prehydrolysate

After acidic steam pretreatment, the solid content and the prehydrolysate were separated. The pretreated solid content contained a total dry matter of 28.7% (*w*/*w*). Corn stover usually contains 33% to 40% cellulose, 13% to 31% hemicellulose, and 5% to 26% lignin [4]. As a consequence of hemicellulose solubilization, high xylose (11.39 g/L), arabinose (1.37 g/L), and galactose (1.35 g/L) were detected, and different degradation products were identified and quantified as soluble compounds in prehydrolysate. Among which, lignin, furfural, 5-hydroxymethylfurfural (5-HMF), and acetic acid were formed at highest concentrations (Table 4). Furfural and 5-HMF are derived from pentoses especially xylose and hexose degradation, respectively, and acetic acid is formed by the hydrolysis of acetyl groups in hemicellulose [38]. In addition, phenolic compounds at a final concentration of 28.6 mM were detected in the prehydrolysate. These compounds, derived from guaiacyl propane units and syringylpropane units present in lignin [39], are considered to be inhibitors and can affect biochemical pathways in the fermenting microorganisms and interact with cellulolytic enzymes, leading to reduced final ethanol titers and volumetric productivities [2-4].

**Table 4:** **Composition of pretreated corn stover and prehydrolysate**

**Corn stover**		**Prehydrolysate**			
**Component**	**% weight**	**Monosaccharides**	**Concentration (g/L)**	**Inhibitors**	**Concentration**
Cellulose	23.15	Glucose	5.74	Furfural	2.28 g/L
Hemicellulose	13.44	Xylose	11.39	5-HMF	0.33 g/L
Lignin	10.92	Arabinose	1.37	Acetic acid	0.309 g/L
Extracts	34.50	Galactose	1.35	Formic acid	0.045 g/L
Unknown compounds	17.99			Lignin	4.9 g/L
				Total phenol	28.6 mM

**Figure 5:**
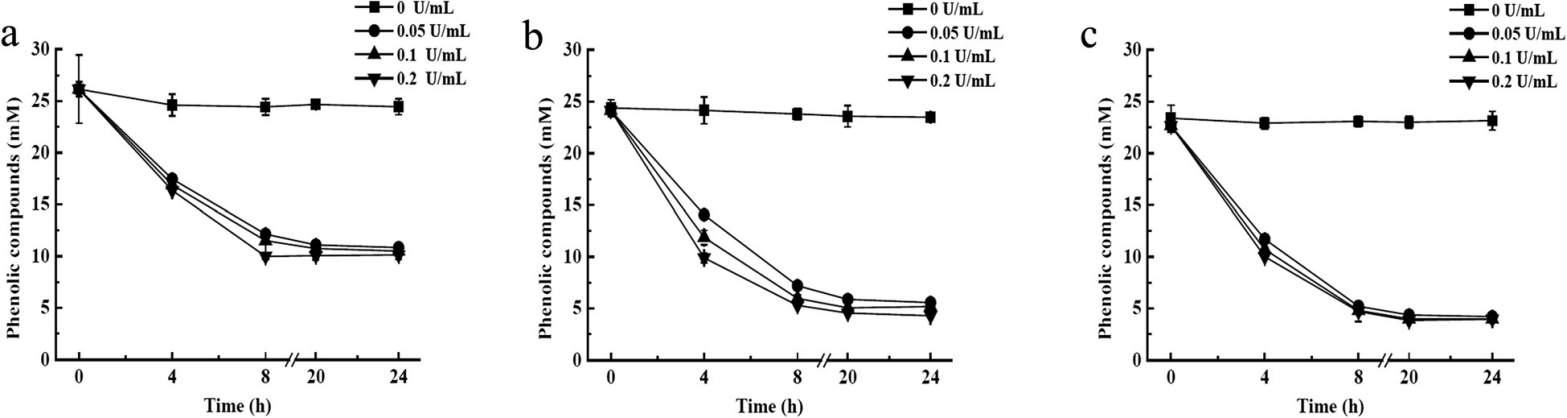
**Time course of phenolic content of prehydrolysate during pretreatment with Glac15 at different pHs. (a)** pH 3.0, **(b)** pH 4.0, and **(c)** pH 5.0.

Laccases have been applied in a few cases to remove specifically phenolic compounds in steam-exploded biomass [7]. However, little information of laccases from *G. lucidum* on removing phenolic compounds from steam-exploded biomass has been observed [7,13]. Our results showed that Glac15 can function on the phenolic compounds derived from steam-exploded corn stover. When prehydrolysate pH was adjusted from 1.4 to 3.0, 4.0, and 5.0 in control samples, total phenolic compound contents decreased from 28.61 to 25.21, 24.39, and 23.38 mM, respectively, in the first 10 min and were not significantly changed even after 24 h incubation, suggesting that phenolic compounds during steam explosion are solubilized and stable at pH 1.4 [5]. However, when pH was increased to 3.0 to 5.0, acetic acid was transformed to acetate. This might lead to the polymerization of phenolic compounds. Addition of Glac15 removed about 60%, 78%, and 84% of the phenolic compounds at pH 3.0, pH 4.0, and pH 5.0, respectively, after 24 h treatment (Figure 5). As lignin derivates generated during pretreatment are phenolic compounds similar to guaiacol and syringaldazine, the increased reduction ability of Glac15 with increment of pH could be explained by the higher laccase activity observed with phenolic substrates at pH 4.5 to 5.0 (Table 3). On the other hand, higher laccase doses (0.05, 0.1, and 0.2 U/mL) did not result in significantly higher detoxification (Figure 5). Increasing enzyme specificity for removing phenols allows decreasing enzymes doses. Therefore, low Glac15 concentrations are suitable for detoxification of steam-exploded corn stover, which is an important aspect for the application of enzymes in industrial applications [5].

**Figure 6:**
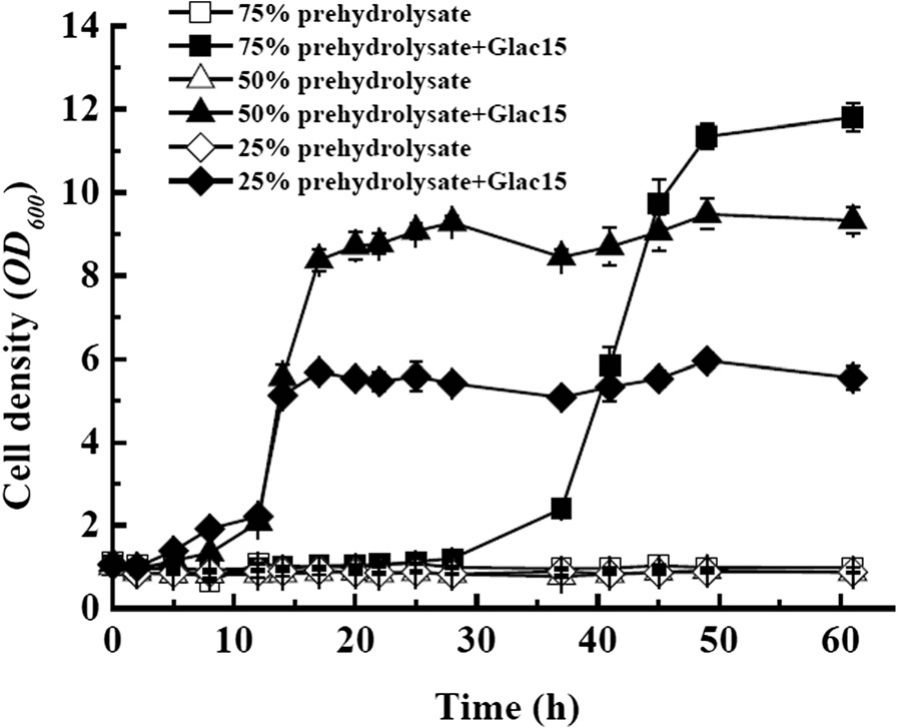
**Time course of yeast growth in the presence of different concentrations of prehydrolysate treated or not with Glac15.**

Glac15-treated prehydrolysate was employed to test the effect of laccase treatment on yeast growth. When *Saccharomyces cerevisiae* CICC31014 strains were cultured in yeast nitrogen base (YNB) medium in the presence of 25%, 50%, and 75% prehydrolysate (the final glucose concentration was 21.44, 22.87, and 24.31 g/L, respectively), strains showed no growth in all of the untreated samples (Figure 6). In contrast, after treatment with laccase, yeast cell growth improved considerably. The biomass reached 5.55 ± 0.27, 9.33 ± 0.32, and 11.81 ± 0.33 (optimal density at 600 nm (OD_600_)) in the samples containing 25%, 50%, and 75% prehydrolysates, respectively. In accordance with Moreno *et al.* [40], who reported the treatment of slurry with laccase increased the yeast cell viability, our results also showed the increased cell viability, evidenced the detoxication effect of Glac15 on prehydrolysate. The higher cell density could be attributed to the higher concentration of glucose added to the medium with the addition of prehydrolysates. On the other hand, the higher the concentration of prehydrolysate, the higher the concentrations of other inhibitors such as furan derivates and weak acids. As a result, yeast growth in the former two samples had a shorter lag period (12 h) than that in the last sample (37 h) (Figure 6).

### Effects of laccase treatment on enzymatic hydrolysis and ethanol fermentation

**Figure 7:**
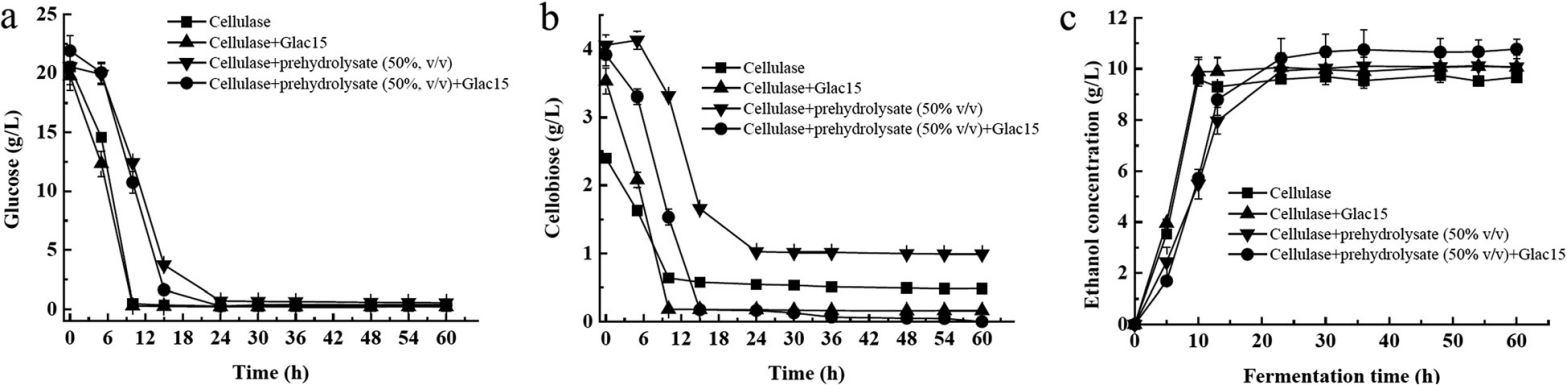
**Time course of sugar consumption and ethanol yield using solid fraction and solid fraction with prehydrolysate (50%,** ***v/v*** **) treated or not with Glac15. (a)** Glucose. **(b)** Cellobiose. **(c)** Ethanol concentration.

The effect of laccase treatment on enzymatic hydrolysis was evaluated. After treating the solid fraction with Glac15, the glucose recovery was not apparently changed when checked by a statistical analysis (Figure 7, Table S2). This is different from the previous studies reported by Tabka *et al.* [41] and Jurado *et al.* [5], who described a lower glucose recovery after enzymatic hydrolysis of steam-exploded wheat straw treated with laccase. The lower recovery was explained by the release of certain phenolic compounds by laccases inhibiting cellulolytic enzymes [5,41,42]. Based on our results, cellobiose was increased from 2.41 ± 0.17 to 3.25 ± 0.21 g/L after treating with Glac15, indicating that cellobiose was accumulated during the process. On the contrary, improved glucose recovery by laccase treatment of steam-exploded softwood has been reported, and a decrease in the unproductive binding of cellulases to lignin after laccase treatment has been suggested [43]. With the addition of prehydrolysate, cellulase activities may have been inhibited, as there was no increase in glucose concentration after addition of prehydrolysate. Statistical analysis showed that the glucose and cellulase recoveries were not significantly changed even after treating with Glac15 (*P* > 0.05), indicating that Glac15 had no effect on saccharification in the presence of prehydrolysate.

From an economical and environmental point of view, the use of prehydrolysate can increase the fermentable sugar concentration, decrease both operational costs and wastewater, and obtain a higher ethanol production [42,44,45]. As a result, four groups containing only solid content (5%, *m*/*v*), solid content (5%, *m*/*v*) and treated with Glac15, solid content (5%, *m*/*v*) containing 50% prehydrolysate, and solid content (5%, *m*/*v*) containing 50% prehydrolysate treated with Glac15 were employed to evaluate the effect of laccase treatment on ethanol fermentation. Fermentation was improved after detoxification with Glac15. After 60 h fermentation with *S. cerevisiae*, results showed that sugar consumption and ethanol yield were higher in samples treated with laccases than in control samples (Figure 7). The final ethanol concentrations of three groups were 9.74 ± 0.18, 10.05 ± 0.15, 10.11 ± 0.21, and 10.81 ± 0.23 g/L, respectively (Figure 7c). Laccase treatment to increase ethanol production has been reported by others. For example, Jonsson *et al.* [46] reported that ethanol productivity was increased by two to three times when treated a willow hemicellulose hydrolysate from willow with laccase and lignin peroxidase from the white-rot basidiomycete fungus *T. versicolor*. When laccase was used to detoxify water-impregnated wheat straw or acid-impregnated wheat straw, ethanol yield was two times higher than the control that has not been treated with laccase [5]. Compared to these results, an increasement of about 10% ethanol was obtained when treated with Glac15. To maximize the improvement of ethanol production caused by laccase treatment, further researches on decreasing the quantity of cellulases enzymes in enzymatic hydrolysis assays to lower activities, or increasing solid fraction consistency, are to be performed.

## Conclusions

In this study, *G. lucidum* 77002 produces 141.1 ± 0.2 U/mL laccase activity within 6 days when using wheat bran and peanut powder as energy sources in liquid culture medium. A new isoenzyme named Glac15 was identified, purified, and characterized. It showed high activity toward phenolic and non-phenolic substrates syringaldazine and ABTS. When used in bioethanol production process, low amount Glac15 removed 84% of the phenolic compounds in prehydrolysate at pH 5 and considerably improved yeast growth. Addition of Glac15 before cellulase hydrolysis improved 10% ethanol yield. Our results showed that the *G. lucidum* laccase Glac15 has potentials in bioethanol production industry.

## Methods

### Chemicals, strains, media, and culture conditions

Filamentous fungus *G. lucidum* 77002 was preserved at culture collection of the School of Life Sciences, Anhui University, China (China Center for Type Culture Collection No. AF2013025). *S. cerevisiae* was from China Center of Industrial Culture Collection, Beijing, China (No. CICC31014). Syringaldazine, 2,6-DMP, guaiacol, catechol, L-dopamine, ABTS, and K_4_Fe(CN)_6_ were purchased from Sigma-Aldrich (St. Louis, MO, USA). Wheat bran was from Zhangyuan (Bozhou, China), and peanut powder was purchased from Huiyou (Hefei, China).

*G. lucidum* 77002 was maintained on potato dextrose agar (PDA, contained 20.0 g glucose, 1.0 g KH_2_PO_4_, 1.5 g MgSO_4_ · 7H_2_O, 50.0 μg vitamin B_1_, 15.0 g agar powder, and 200.0 g potato extract liquid/L) slants at 4°C. YMG liquid medium (contained 10.0 g malt extract, 4.0 g yeast extract, and 10.0 g glucose/L) was used to prepare *G. lucidum* 77002 inoculation. The liquid fermentation medium used for producing laccase contained 30.0 g peanut powder, 30.0 g wheat bran, and 1.5 g KH_2_PO_4_/L. YNB medium (contained 1.7 g yeast nitrogen base, 5.0 g ammonia sulfate, and 20.0 g glucose/L) was used for culture of *S. cerevisiae*.

### Laccase production in liquid culture

When producing laccases, four to five plugs (diameter in 5 mm) of *G. lucidum* 77002 actively grown on PDA plates were used to inoculate 250-mL Erlenmeyer flasks containing 100 mL YMG medium. The culture was incubated at 28°C on a rotary shaker at 120 rpm. A 4-day-old liquid culture was homogenized with a sterile blender at 3,000 rpm for 10 s, and a volume of 5 mL was inoculated into the new 400 mL (in 1-L Erlenmeyer flasks) liquid fermentation medium and was cultivated under the same conditions for 8 days.

### Laccase purification

Unless otherwise stated, all procedures were performed at 4°C. The culture fluid was first filtered through six layers of sterile gauze and washed with three volumes of citrate-phosphate buffer (50 mM, pH 5.0). The aqueous solution (1.25 L) was centrifuged at 10,000 × *g* for 10 min and then concentrated to 100 mL in a Minitan Ultrafiltration System (Millipore, Bedford, MA, USA) with a low-binding regenerated cellulose membrane (Millipore, Bedford, MA, USA). The concentrate was centrifuged at 12,000 × *g* for 20 min to discard the sediment generated during the ultrafiltration, and the supernatant was then dialyzed against buffer A (20 mM citrate-phosphate, pH 7.5) overnight followed by centrifugation as before. The supernatant was applied to a DEAE-Sepharose FF column (10 × 200 mm, Amersham Pharmacia, Uppsala, Sweden) preequilibrated with buffer A. The column was then washed with approximately 100 mL buffer A to remove melanin and polysaccharide and eluted with a linear gradient of (NH_4_)_2_SO_4_ (0 to 1 M in buffer A; flow rate 0.8 mL/min). The fractions containing laccase were pooled and dialyzed against 50 mM citrate-Na_2_HPO_4_ buffer (pH 7.5) and stored at 4°C.

### Laccase identification

The purified laccase was identified according to Rühl *et al.* [47] with slight modifications. Briefly, the purified protein was recovered for SDS-PAGE [48]. Coomassie brilliant blue R-250-stained protein bands were cut with a razor blade. The gel pieces were digested with trypsin and analyzed by LC-ESI-MS/MS (LTQ, Thermo Fisher Scientific, Shanghai, China). Proteins were then identified by searching the data against a database of the *G. lucidum* Xiangnong No.1 genome [17]. To further confirm the identity of protein, purified laccase was electro-blotted directly from the SDS-PAGE gel onto a polyvinylidene difluoride membrane (Sequi-Blot PVDF, Bio-Rad, Hercules, CA, USA) and located by Coomassie blue R-250 staining. The band on the PVDF membrane corresponding to the Glac15 was excised and subjected to Edman degradation. N-terminal sequences of the proteins were determined using a PPSQ-33A Edman degradation unit (Shimadzu, Kyoto, Japan) and the data was processed by PPSQ-30 Data Processing software.

### Enzyme assay

The assay mixture consisted of 10 μL of appropriately diluted laccase stock and 990 μL of 50 mM citrate-phosphate buffer (pH 4.0) containing 0.5 mM ABTS (*ε*_420_ = 36,000 M^-1^ cm^-1^) [49]. The reaction was initiated by adding enzyme into the solution. After incubation at 50°C for 3 min, the absorbance was immediately measured at 420 nm. Alternative substrates for the measurement of laccase activity were 100 μM syringaldazine (*ε*_525_ = 65,000 M^-1^ cm^-1^) [50], 2 mM 2,6-DMP (*ε*_468_ = 49,600 M^-1^ cm^-1^) [51], 2 mM guaiacol (*ε*_465_ = 12,000 M^-1^ cm^-1^) [52], 2 mM catechol (*ε*_388_ = 1,300 M^-1^ cm^-1^) [51], 2 mM L-dopamine (*ε*_475_ = 2,835 M^-1^ cm^-1^) [53], and 2 mM K_4_Fe(CN)_6_ (*ε*_405_ = 900 M^-1^ cm^-1^) [54], respectively. Reactions with heat-treated laccase were used as the controls. One activity unit (U) was defined as the amount of enzyme required for oxidizing 1 μmol of substrate/min [33].

### Laccase characterization

The effect of pH on laccase activity was examined in the pH range of 2.5 to 7.0 at 50°C in 50 mM citrate-phosphate buffer. The enzyme stability against pH was determined by measuring the residual laccase activities after incubation at 50°C in the aforementioned buffers, and residual activities were determined every 15 min. The effect of temperature on the activity was measured by incubating protein at optimal pH and a temperature range of 30°C to 80°C in an interval of 5°C. Thermostability was determined by incubating protein at various temperatures (30°C to 70°C) at pH 5.0, and residual activities were determined every 10 min.

The effects of metal ions (Cu^2+^, Mn^2+^, Li^+^, Ca^2+^, Zn^2+^, K^+^, using sulfate as the donor, 0 to 50 mM), SDS (0 to 50 mM), EDTA (0 to 50 mM), and organic solvents (ethanol, dichloromethane, ethyl ether, ethyl acetate, DMSO, acetaldehyde, and acetone, 0 to 25%, *v*/*v*) on the enzyme activity were investigated by adding different concentrations of each effector into the reaction system when addition of substrate ABTS. The enzymatic assays were conducted under the conditions described in enzyme assay.

### Enzyme kinetics

The kinetics and the specificity of Glac15 toward each of these substrates were measured at the optimum pH of the enzyme (Table 3). The Michaelis-Menten and catalytic constants (*K*m, *k*_cat_, and *k*_cat_/*K*m) were determined by incubating Glac15 with various concentrations of syringaldazine, 2,6-DMP, guaiacol, catechol, L-dopamine, ABTS, and K_4_Fe(CN)_6_. The values for the kinetic parameters and their corresponding errors were then calculated.

### Corn stover pretreatment

Corn stover was used as raw material. It was milled using a laboratory hammer mill, in order to obtain a chip size round 2 mm, and stored at room temperature until use. Milled biomass was pretreated by steam explosion with a raw material: H_2_O proportion of 1:10 (*w*/*v*) and in the presence of 0.8% H_2_SO_4_ (*v*/*v*) in a 10-L reactor at 160°C for 30 min. After steam explosion treatment, the whole slurry was filtered with the aim of obtaining a liquid fraction and a solid fraction.

### Laccase treatment of the prehydrolysate

The effect of laccase treatment on phenolic compounds in corn stover prehydrolysate was evaluated using the liquid fraction obtained from pretreatment. Before adding Glac15, prehydrolysate was adjusted to pH 3.0 to pH 5.0 and kept for 1 h and then incubated with 0.05, 0.1, or 0.2 U/mL of purified Glac15 for 24 h at 45°C in a rotary shaker (200 rpm) [5]. Samples were periodically taken and the supernatants analyzed for total phenols as described below. To evaluate the effect of laccase treatment on yeast growth, prehydrolysates were diluted to 75%, 50%, and 25% of original concentration by 4× YNB medium (the final concentration of YNB medium was 1×) [55]. After treatment with 0.05 U/mL Glac15 at pH 5.0 and 45°C for 8 h, *S. cerevisiae* in a final concentration of 1 OD_600_ were inoculated and were incubated at 30°C in a rotary shaker (200 rpm) for 60 h. In all cases, control assays were performed under the same conditions but without addition of laccase Glac15. All the experiments were carried out in triplicate.

### Laccase treatment and enzymatic hydrolysis of thermochemical pretreated corn stover

Laccase treatment on pretreated solid fraction was performed as follows: in the first set of experiments, a total volume of 40 mL containing 2.0 g (dry weight, 5% *w*/*v*) pretreated solid fraction, 2.0 g/L (NH_4_)_2_SO_4_, 4.0 g/L KH_2_PO_4_, 1.0 g/L MgSO_4_, and 0.2 g/L CaCl_2_ was used; in the second set of experiments, 5% solid fraction and 50% prehydrolysate (*v*/*v*) were added into the medium described above (the total volume was also 40 mL). Depending on the previous experiments, an enzyme loading of 0.05 U/mL of Glac15 was added and then incubated at 45°C and pH 5.0 in a rotary shaker (200 rpm). Control assay was performed under the same condition without addition of laccase. All experiments were carried out in triplicate. After 8 h of treatment, another 24-h enzymatic hydrolysis at 45°C was performed following the addition of 30 FPU/g (dry weight) cellulase (Sunson Biotech., Peking, China). Then the temperatures of hydrolyzed samples were reduced to 30°C and *S. cerevisiae* in a final concentration of 1.0 OD_600_ was aseptically inoculated. The fermentation was performed at 30°C in a rotary shaker (200 rpm) for another 60 h. Samples of different time points were recovered and centrifuged at 15,000 × *g* for 5 min; the supernatants were filtered through a 0.22-μm Millipore filter (Millipore, Bedford, MA, USA) and analyzed for glucose consumption and ethanol concentration.

### Analytical methods

Biomass was determined using absorbance at 600 nm. The ethanol concentration was determined using an HPLC system according to Ji *et al.* [55]. Xylose, arabinose, galactose, furfural, 5-HMF, formic acid, and acetic acid concentrations were measured according to Moreno *et al.* [42]. Total phenols were measured according to Folin-Ciocalteu method using vanillin as a standard [56]. The data presented are the average values from triplicate technical repeats of measurements.

## Additional file

Additional file 1: Tables S1 and S2
**Table S1:** **Laccase peptides detected by LC-ESI-MS/MS and database search.** Table S2: Sugar and ethanol concentrations after treated in different ways.

## Abbreviations

2,6-DMP: 2,6-dimethoxyphenol
5-HMF: 5-hydroxymethylfurfural
ABTS: 2,2′-azino-bis-(3-ethylbenzothiazoline-6-sulfonic acid)
DMSO: dimethyl sulfoxide
EDTA: ethylenediaminetetraacetic acid
OD_600_: optimal density at 600 nm
PAGE: polyacrylamide gel electrophoresis
PDA: potato dextrose agar
SDS: sodium dodecyl sulfate
