# Supplementary material for: Identification of a laccase Glac15 from Ganoderma lucidum 77002 and its application in bioethanol production
Source: Biotechnol Biofuels. 2015 Mar 31;8:54. doi: 10.1186/s13068-015-0235-x (PMC4399389; doi:10.1186/s13068-015-0235-x)
Supplement: Additional file 1: Tables S1 and S2 — Table S1: Laccase peptides detected by LC-ESI-MS/MS and database search. Table S2: Sugar and ethanol concentrations after treated in different ways. [file 13068_2015_235_MOESM1_ESM.docx]

Table S1 Laccase peptides detected by 1D-LC-ESI-MS/MS and database search.

| **Protein** | **Reference**  **Scan(s)** | **Peptide** | **MH+** | **P (pro)**  **P (pep)** | **Score**  **XC** | **Coverage**  **DeltaCn** | **MW**  **Sp** | **Peptide (Hits)**  **Ions** |
| --- | --- | --- | --- | --- | --- | --- | --- | --- |
| **Glac15** |  |  |  | 7.77E-16 | 40.31 |  | 48516.9 | 16 (16 0 0 0 0) |
|  | 7769 | K.TTSIHWHGFFQK.G | 1488.73826 | 1.98E-08 | 3.72 | 0.80 | 1194.9 | 17/22 |
|  | 9140 | R.AAVVVNGVFPGPLITGNMGDR.F | 2084.09572 | 1.61E-03 | 3.76 | 0.76 | 934.0 | 22/40 |
|  | 9347 | R.ANPNFGNVGFTDGINSAILR.Y | 2077.04613 | 1.90E-14 | 5.50 | 0.96 | 1836.2 | 34/76 |
|  | 9157 | R.ANPNFGNVGFTDGINSAILR.Y | 2077.04613 | 7.77E-16 | 6.22 | 0.91 | 1483.1 | 27/38 |
|  | 3358 | R.ATPGNPTQGGVDK.A | 1241.61206 | 1.40E-10 | 2.63 | 0.63 | 596.2 | 21/24 |
| **Glac06** |  |  |  | 7.77E-16 | 30.31 |  | 55225.5 | 20 (20 0 0 0 0) |
|  | 9347 | R.ANPNFGNVGFTDGINSAILR.Y | 2077.04613 | 1.90E-14 | 5.50 | 0.96 | 1836.2 | 34/76 |
|  | 9157 | R.ANPNFGNVGFTDGINSAILR.Y | 2077.04613 | 7.77E-16 | 6.22 | 0.91 | 1483.1 | 27/38 |
|  | 6455 | R.DVVSTGTPAAGDNVTIR.F | 1672.85005 | 2.55E-07 | 4.38 | 0.90 | 1226.7 | 23/32 |
|  | 7497 | R.SAGSTVYNYDNPVWR.D | 1728.79762 | 2.20E-07 | 4.76 | 0.75 | 1159.4 | 19/28 |
|  | 7457 | R.SAGSTVYNYDNPVWR.D | 1728.79762 | 1.10E-04 | 5.04 | 0.77 | 2417.1 | 33/56 |

Table S2 Sugar and ethanol concentrations after treated in different ways.

| Assay groups | Glucose (g/L) | Cellobiose (g/L) | Ethanol (g/L) |
| --- | --- | --- | --- |
| solid content (5%, m/v) | 20.53±0.87 | 2.41±0.17 | 9.74±0.18 |
| solid content (5%, m/v) + Glac15 | 19.76±1.11 | 3.25±0.21 | 10.05±0.15 |
| solid content (5%, m/v)+ prehydrolysate (50%, v/v) | 20.54±0.43 | 4.06±0.11 | 10.11±0.21 |
| solid content (5%, m/v)+ prehydrolysate (50%, v/v)+ Glac15 | 21.90±1.29 | 3.92±0.19 | 10.81±0.23 |
